# Cardiac magnetic resonance imaging for myocardial perfusion and diastolic function - reference control values for women

**DOI:** 10.1186/1532-429X-15-S1-P118

**Published:** 2013-01-30

**Authors:** Puja K Mehta, Afsaneh Haftbaradaran, Megha Agarwal, Behzad Sharif, David Chen, Chrisandra Shufelt, Edward B Gill, Greg Lentz, Daniel S Berman, Margo Minassian, Piotr Slomka, Debiao Li, Noel Bairey Merz, Louise E Thomson

**Affiliations:** 1 Women Heart Center, Cedar sinai medical center, Los Angeles, CA, USA

## Background

Diastolic dysfunction and angina in the absence of obstructive coronary artery disease are more common in women. Microvascular coronary dysfunction (MCD) and are associated with an adverse cardiovascular prognosis. Cardiac Magnetic resonance imaging (CMRI) is established for assessment of left ventricular (LV) systolic function however but has not been wildly used to assess diastolic function is unknown. Stress CMRI may be used for measurement of myocardial perfusion reserve index (MPRI) and LV diastolic function using routine imaging techniques, which may be particularly relevant for detection of MCD in women. Normal reference values for MPRI and LV diastolic function in asymptomatic middle-aged, overweight women have not previously been established.

## Methods

Twenty one women age and BMI-matched to a prior MCD population (Table 1) and free of clinical cardiovascular disease or cardiovascular risk factors were screened with normal maximal Bruce protocol exercise treadmill testing and underwent CMRI (1.5 Tesla) using a standardized protocol of adenosine stress and rest perfusion and LV cine imaging. CASS MRV software (Pie Medical®) was used for calculation of MPRI, ventricular volumetric filling profiles, and ejection fraction.

**Table 1:** 

Cardiac Magnetic Resonance Imaging	(n=21) (mean ± SD)
MPRI (Myocardial Perfusion Reserve Index)	2.19 + 0.38

Peak filling rate	366.5 ± 75.2 ml/s

Peak filling rate (adjusted for end diastolic volume)	2.9 ± 0.4 ml/s

Peak filling rate (adjusted for stroke volume)	4.2 ± 0.6 ml/s

Time to peak filling rate	200.0 ± 19.7 ms

End diastolic volume/ end diastolic volume index	128.6 ± 27.7/ 73.0 ± 14.5 ml

End systolic volume/ end systolic volume index	36.8 ± 7.9/ 20.9 ± 4.0 ml

Left ventricular ejection fraction	70.4 ± 3.9%

## Results

Mean age was 51.1 ± 8.3 yrs and mean BMI was 25.1 ± 3.8 kg/m^2^. Reference control values for MPRI and LV diastolic filling are shown in the table.

## Conclusions

Automated CMRI segmentation can provide quantitative perfusion and LV diastolic function profiles that may offer insight into MCD. We report normal values for MPRI, and LV diastolic filling by CMRI in a reference group of asymptomatic, overweight middle-aged women. These data can be used in comparisons to suspected and known MCD and diastolic dysfunction populations.

## Funding

This work was supported by contracts from the National Heart, Lung and Blood Institutes, nos. N01-HV-68161, N01-HV-68162, N01-HV-68163, N01-HV-68164, a GCRC grant MO1-RR00425 from the National Center for Research Resources, and grants from the Gustavus and Louis Pfeiffer Research Foundation, Denville, New Jersey, the Women's Guild of Cedars-Sinai Medical Center, Los Angeles, California, the Edythe L. Broad Women's Heart Research Fellowship, Cedars-Sinai Medical Center, Los Angeles, California, and the Barbra Streisand Women's Cardiovascular Research and Education Program, Cedars-Sinai Medical Center, Los Angeles.

